# Supplementary material for: The ALFA-tag is a highly versatile tool for nanobody-based bioscience applications
Source: Nat Commun. 2019 Sep 27;10:4403. doi: 10.1038/s41467-019-12301-7 (PMC6764986; doi:10.1038/s41467-019-12301-7)
Supplement: Supplementary file 1 — Supplementary Information [file 41467_2019_12301_MOESM1_ESM.pdf]

Supplementary Information

The ALFA-tag is a highly versatile tool for nanobody-based bioscience applications

Götzke et al.

Supplementary Figures

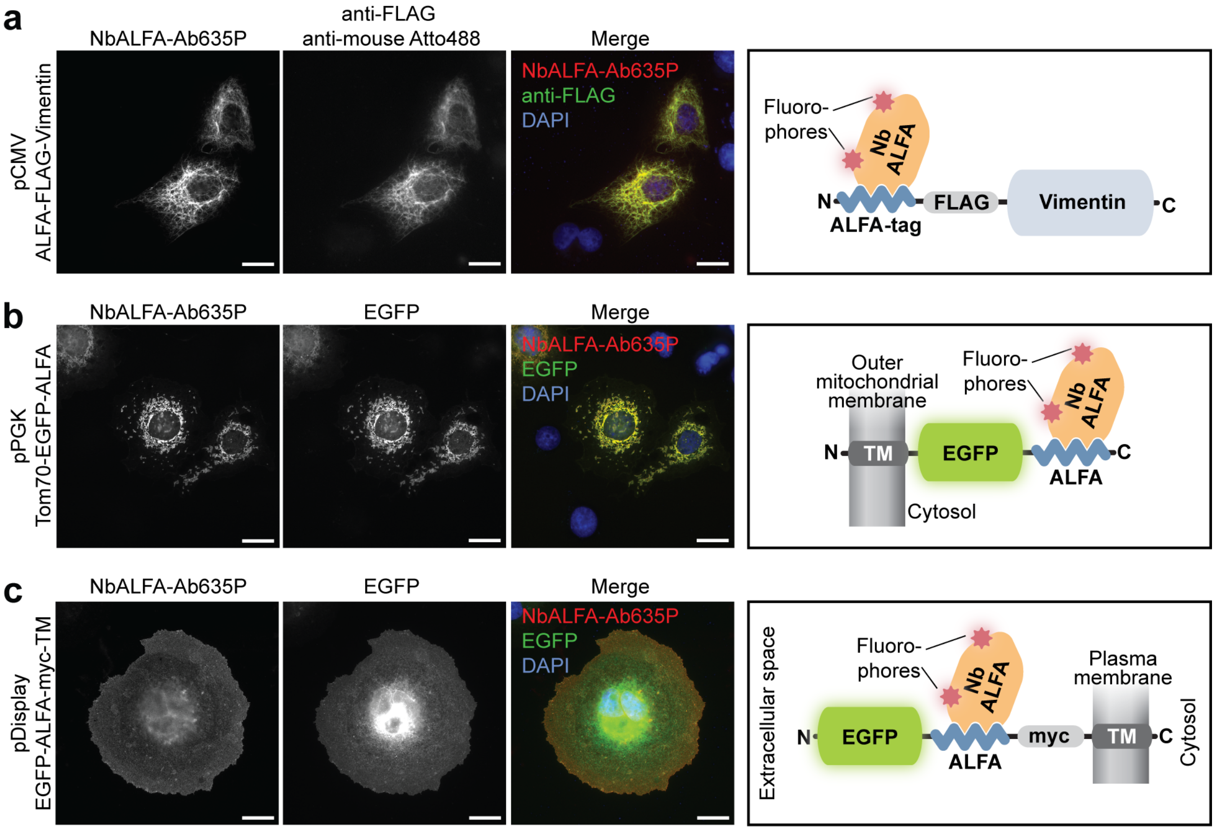

Supplementary Figure 1 – related to Figure 1: ALFA-tagged proteins can be detected by fluorescently labeled NbALFA regardless of the localization of the tag within the fusion protein.

COS-7 cells were transfected with constructs encoding proteins fused to an ALFA-tag at their N-terminus (ALFA-FLAG®-Vimentin; **a**) C-terminus (Tom70-EGFP-ALFA; **b**), or within individual protein-domains (EGFP-ALFA-myc-TM; **c**). Cells were fixed with 4% PFA and stained as indicated. For experiments shown in panels (**a**) and (**b**), cells were permeabilized with 0.1% TritonX-100; for panel (**c**), cells were stained under non-permeabilizing conditions. TM: transmembrane domain. Scale bars: 20µm. Sketches illustrate the topology of substrates and detection by fluorescently labeled NbALFA (NbALFA-Ab635P). Colors scheme used for sketches: NbALFA (orange), ALFA-tag (blue), GFP (green), fluorophore (red star), FLAG tag (grey), myc tag (grey), vimentin (light blue).

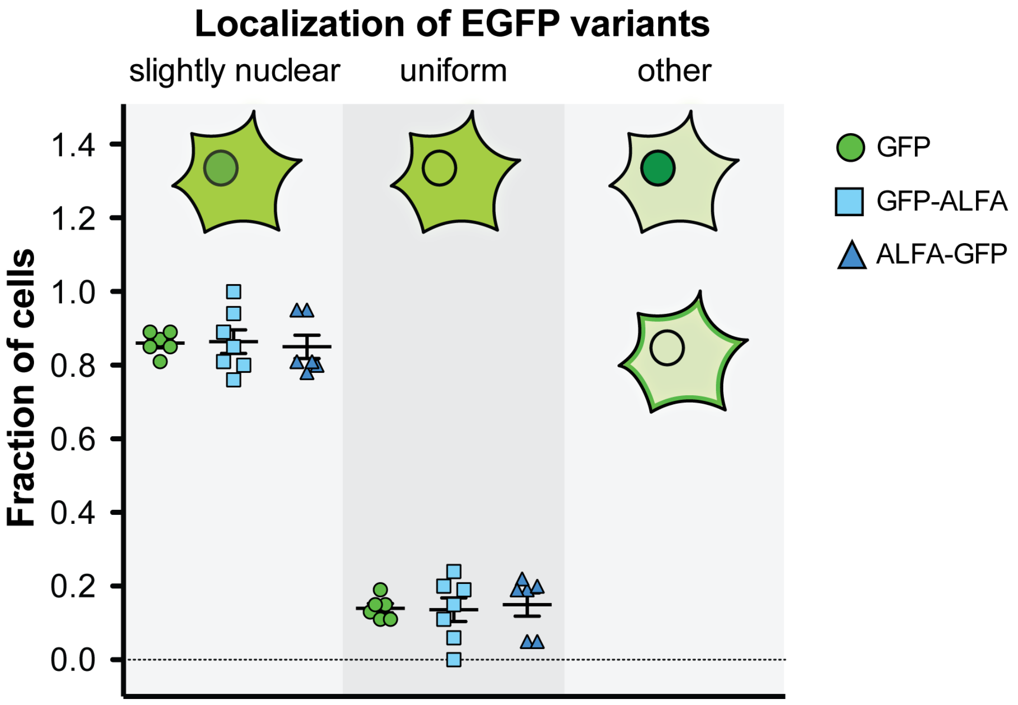

Supplementary Figure 2 – related to Figure 1: N- or C-terminal ALFA-tag fusions show the correct intracellular localization.

3T3 cells were transiently transfected with EGFP fusions harboring N- or C-terminal ALFA tags. Non-tagged EGFP from pEGFP-N1 served as a control. The localization of the respective EGFP variants was analyzed on 6-7 individual images for each construct. Between 120 and 130 cells were imaged per construct and the localization of EGFP was analyzed. In general, each EGFP construct displayed a distribution across the cytosol and the nucleus. Cells were distributed into three groups (slightly nuclear, uniform and other) according to the observed nucleocytoplasmic localization of EGFP. Standard deviations represented by error bars were calculated from values obtained from individual images. Differences between the localizations of tagged and non-tagged EGFP variants were statistically not significant (Student's t-test).

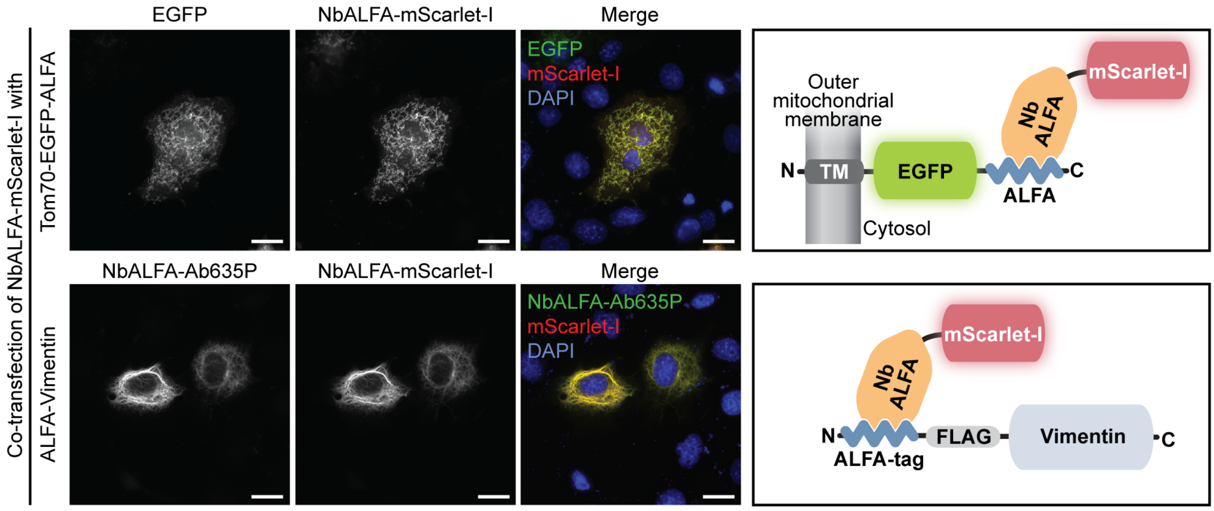

Supplementary Figure 3 – related to Figure 1: Target protein detection using NbALFA expressed the cytoplasm of mammalian cells.

COS-7 cells were co-transfected with an NbALFA-mScarlet-I fusion and indicated ALFA-tagged target proteins. Target proteins were detected either using the intrinsic EGFP fluorescence (TOM70-EGFP-ALFA) or by immunofluorescence using NbALFA-Ab635P (ALFA-Vimentin). In parallel, NbALFA-mScarlet-I was detected by its red fluorescence. The excellent co-localization shows that NbALFA expressed in the cytoplasm of mammalian cells can be used for targeting ALFA-tagged proteins in living cells. Scale bars: 20µm. Colors scheme used for sketches: NbALFA (orange), ALFA-tag (blue), GFP (green), mScarlet-I (red), FLAG tag (grey), vimentin (light blue). The lower panel recapitulates data shown in Figure 1g and is repeated here for a better comparison.

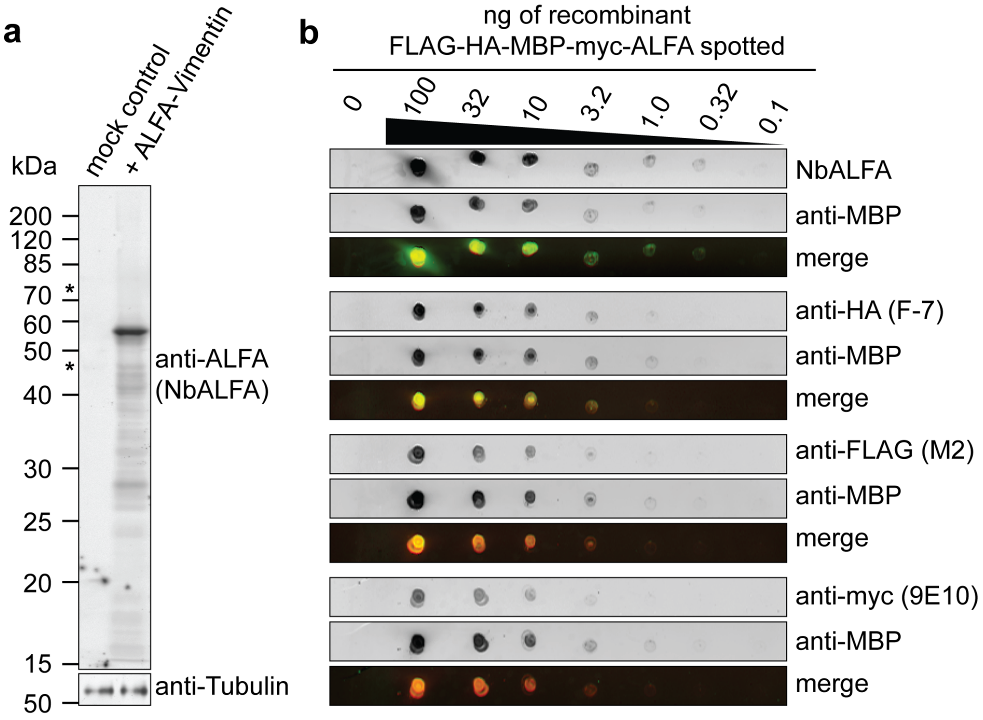

Supplementary Figure 4 – related to Figure 2: Highly sensitive detection of ALFA-tagged target proteins by Western blot using fluorescently labeled NbALFA.

a Complete lanes of the Western blot shown in Figure 2a. Note that in the absence of any vector encoding an ALFA-tagged protein (mock control), only very minor bands (black asterisks) can be detected using NbALFA directly labeled with IRDye800CW. **b** In addition to the data presented in Figure 2c, detection of MBP by a combination of rabbit polyclonal serum recognizing MBP and an anti-rabbit IgG IRDye680RD (Li-Cor #925-68071) is shown as an internal loading control. Individual signals are in gray levels and, colored overlays show MBP signals in red and signals corresponding to epitope tags in green.

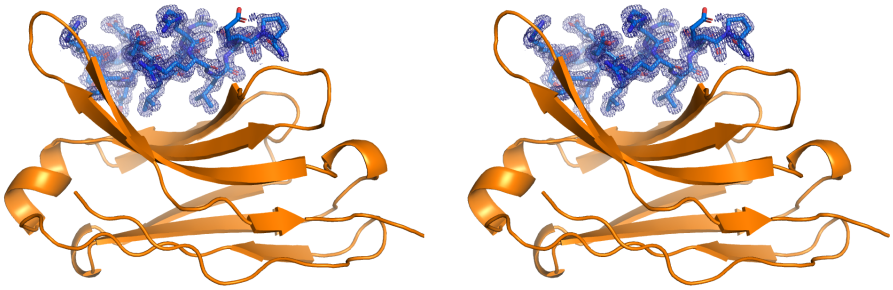

Supplementary Figure 5 – related to Figure 3: Stereo view of NbALFA (orange) in complex with the ALFA-tag (blue).

Electron density of the ALFA-tag is shown in mesh representation ($2F_O$-F_C map contoured at 1.0 σ).

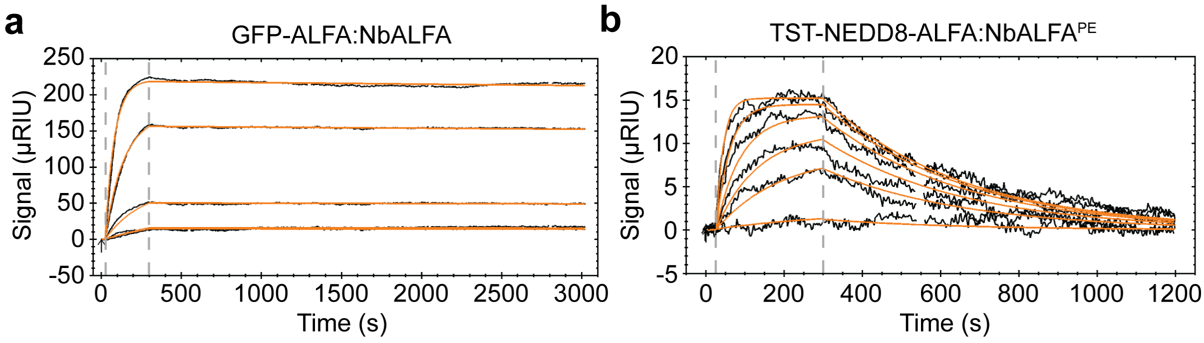

c

Nanobody	Target	k_{on} ($M^{-1}s^{-1}$)	k_{off} (s^{-1})	K_D (nM)
**NbALFA**	GFP-ALFA	3.6 (± 0.1) x 10^{5}	9.4 (± 0.2) x 10^{-6}	0.026 (± 0.001)
**NbALFA^{PE}**	TST-NEDD8-ALFA	2.7 (± 0.4) x 10^{5}	2.9 (± 0.5) x 10^{-3}	11.0 (± 0.6)

Supplementary Figure 6, related to Figure 4: Affinity of NbALFA and NbALFA^{PE} for ALFA-tagged target proteins.

a Representative SPR sensogram for NbALFAST binding to an ALFA-tagged target protein. A serial dilution of analyte (NbALFAST; 50nM, 25nM, 12.5nM, 6.25nM, 3.13nM) was injected over a sensorchip surface coated with GFP-ALFA. Black lines: data recorded; orange lines: fits used to analyze data. **b** Representative SPR sensogram for NbALFA^{PE} binding to an ALFA-tagged target protein. A serial dilution of analyte (NbALFA^{PE}; 200nM, 100nM, 50nM, 25nM, 12.5nM, 6.25nM) was injected over a sensorchip surface coated with TST-NEDD8-ALFA. Black lines: data recorded; orange lines: fits. **c** Summary of affinity data obtained from 3 (NbALFA) or 4 (NbALFA^{PE}) independent experiments, respectively. Standard deviations are given in brackets.

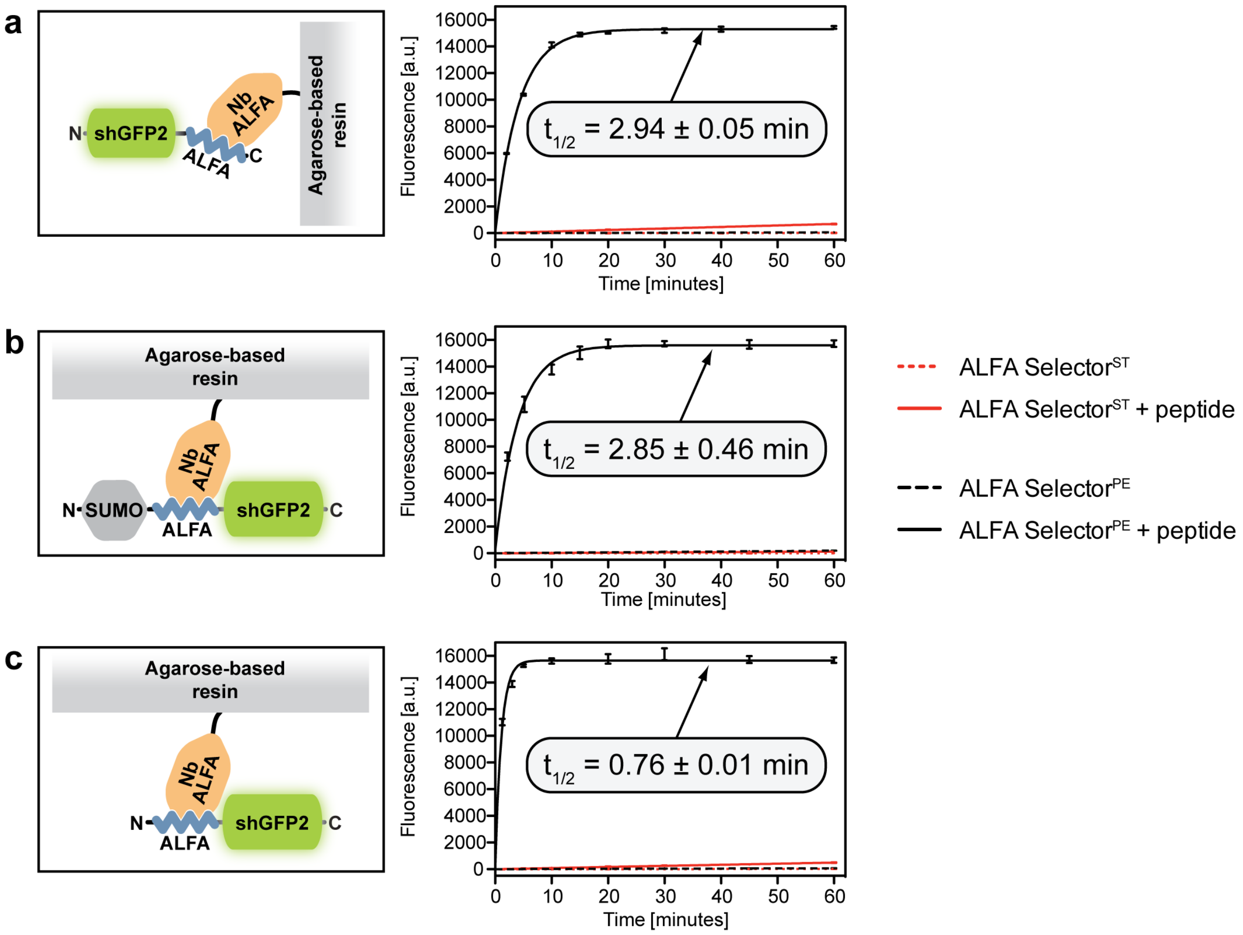

Supplementary Figure 7 – related to Figure 4b: Peptide elution of ALFA-tagged GFPs from ALFA Selector resins.

20µL ALFA SelectorST (presenting NbALFA) or ALFA Selector^{PE} (presenting NbALFA^{PE}) were charged with shGFP2-ALFA (**a**), bdSUMO-ALFA-shGFP2 (**b**) or ALFA-shGFP2 (**c**) as examples of C-terminal, in between folded domains and N-terminal ALFA-tagged POI respectively. After washing with PBS, the beads were suspended in a 10-fold excess of PBS containing 200µM free ALFA peptide and gently mixed at 25°C. Control reactions were carried out without peptide. At indicated time points, specific elution from the beads was quantified using the GFP fluorescence released into the supernatant. Shown are mean fluorescence readings of three experiments. Error bars represent standard deviations for each time point. Lines represent fits to a single exponential. Half times are given for peptide elution from ALFA Selector^{PE} only. For all substrate proteins, peptide elution from ALFA SelectorST was inefficient even after prolonged incubation. In the absence of ALFA peptide, the ALFA-tagged target proteins remained tightly bound to both resins. Note that peptide elution of shGFP2 harboring an N-terminal ALFA tag from ALFA Selector^{PE} is quicker as compared to substrates harboring the ALFA tag at internal or C-terminal positions. Panel (**a**) recapitulates data shown in Figure 4b and is repeated here to allow a direct comparison. Colors scheme used for sketches: NbALFA (orange), ALFA-tag (blue), GFP (green), bdSUMO (grey).

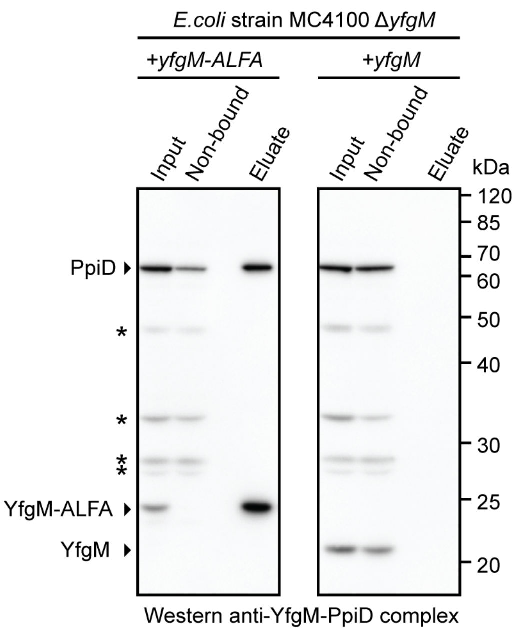

Supplementary Figure 8 – related to Figure 5c: Pull-down of a native *E. coli* YfgM-PpiD inner membrane protein complex using the ALFA Selector (complete blots).

A *yfgM* deletion strain was complemented with either C-terminally ALFA-tagged (left panel) or untagged YfgM (right panel; control reaction) expressed from a low-copy vector. Membrane protein complexes were solubilized from total lysate using DDM. Complexes containing YfgM-ALFA were purified in a single step using ALFA Selector^{PE} affinity resin and eluted under native conditions using 200µM ALFA peptide. Samples corresponding to 1/800 of the input and non-bound material or 1/80 of eluate fractions were resolved by SDS-PAGE and analyzed by Western blot. A rabbit serum raised against the YfgM-PpiD complex recognized both, PpiD and YfgM, in the input fractions. ALFA Selector^{PE} specifically immunoprecipitated the native protein complex comprising ALFA-tagged YfgM and its interaction partner PpiD. In the control reaction (no ALFA tag on YfgM), both proteins were absent in the eluate. The figure recapitulates data shown in Figure 5c, here, however, complete membranes are shown. Asterisks mark non-specific bands recognized by the polyclonal antiserum.

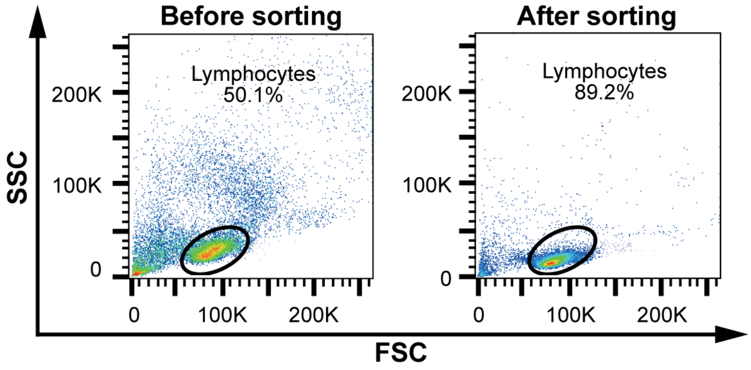

Supplementary Figure 9 – related to Figure 6: Gating strategy to identify lymphocytes.

Total human PBMCs were left untreated (Before sorting) or isolated using an ALFA Selector^{PE} resin loaded with an ALFA-tagged anti-human CD62L nanobody (After sorting) as shown in Figure 6. Forward-scatter/side-scatter plots showing the lymphocyte gate (black oval) that has been used to exclude cellular debris from the analysis. The numbers give the percentage of all events in the lymphocyte gate. Identical gates have been applied to all analyses shown in Figure 6.

Supplementary Table 1: Properties of common epitope tag systems

		FLAG-tag[1]	HA-tag[2]	myc-tag[3]	Twin-Strep-tag[4]	polyHis-tag[5,6]	EPEA/C-tag[7]	Spot-tag[8,9]	ALFA tag
**Properties of tag**	Sequence	DYKDDDDK	YPYDVPDYA	EQKLI-SEEDL	WSHPQFEK-$(GGGS)_2$-GGSA-WSHPQFEK	His_{3-10}	EPEA	PDRVRA-VSHWSS	(P)SRLEEELR RRLTE(P)
	Size (amino acids)	8	9	10	28	3-10	4	12	14-15
	Mass (kD)	1.1	1.1	1.2	2.9	0.4-1.4	0.5	1.4	~1.8
	Charge at pH7.0	-3	-2	-3	0.2	0.3-1	-2	1.1	0
	pKi	3.5	0	3.5	8.4	14	0	12.1	8.1
	Physical size (nm)	2.2	2.5	2.8	>6	0.8-2.8	1.2	3.3	2.0
	Water solubility	+	poor	+	+	poor	+	+	+
	Structured in solution	–	–	–	–	–	-	–	+[10]
	Stable	(+)[11]	(+)[12]	+	+	+	n.d.	+	+
	Resistant to aldehyde modification[(a)]	–	+	–	–	+	+	+	+
	Unique within model organisms	+	–	–	(+) [(b)]	(–) [(c)]	–	(–) [(d)]	+
**Properties of binder**	Name	M1, M2, M5	F-7, 12CA5	9E10	StrepTactin-XT	Ni^{2+}/Co^{2+}-chelate	NbSyn2	Spot-nanobody	NbALFA
	Type	mAb	mAb	mAb	Protein	Inorganic	sdAb	sdAb	sdAb
	Size (kD)	~150	~150	~150	~60	n.a.	~15	~15/30 [(e)]	~15
	No. of polypeptides	4	4	4	4	n.a.	1	1	1
	No of binding sites	2	2	2	2	1	1	1-2 [(e)]	1
	Affinity	n.d.	n.d.	n.d.	~60 pM [(f)]	n.d.[(g)]	>190 nM	~6 nM	26 pM
	Genetically accessible	–	–	–	+	–	+	+	+
**Appli-cations**	Protein purification	+	+	+	+	+	+	+	+
	Imaging	+ [(h)]	+ [(h)]	+ [(h)]	n.d.	(–) [(i)]	–	(+) [(e)]	+
	In-vivo applications	–	–	–	–	–	–	–	+

Abbreviations: n.a.: not applicable; n.d.: no data available; mAb: monoclonal antibody; sdAb: single-domain antibody

(a) Fixation by amine-reactive fixatives and cross-linkers; deduced from sequence
(b) Binder also recognizes biotinylated proteins
(c) Binder recognizes endogenous proteins with multiple accessible histidines.
(d) Binder also recognizes endogenous β-catenin
(e) Binder needs to be dimerized for high-profile imaging applications[8]
(f) https://www.iba-lifesciences.com/tl_files/ProteinProductionAssays/5-Immobilization/DynamicBiosensors-Application-Note-StrepTactinXT-switchSENSE.pdf
(g) Depends on chelate and polyHis-tag used
(h) Multiple tags used in tandem for optimal performance[13–16]
(i) Rarely used for imaging in combination with Tris-NTA derivatives[17]

Supplementary Table 2. Intramolecular side-chain interactions within the ALFA peptide.

Residue [(a)]	Interaction	Distance (Å)
E7'-R10'	Salt bridge	2.67
E7'-R11'	Salt bridge	2.77
R9'-T13'	H-bond	3.07
L4'-L8'-L12'	Hydrophobic array	

[(a)]ALFA peptide residues are marked by an apostrophe.

Supplementary Table 3. List of intermolecular interactions between NbALFA and the ALFA peptide.

ALFA peptide residue [(a)]	NbALFA residue	Interaction	Distance (Å)
S2'	E58	H-bond	2.68
R3'	D105	Salt bridge	3.05
E5'	R59	Salt bridge	2.71
E5'	N61	H-bond	2.81
R11'	D112	Salt bridge	2.71
R11'	Y42	H-bond	2.98
E14'	R65	Salt bridge	2.80
R3'	F110	Cation-Pi interaction[18]	

[(a)]ALFA peptide residues are marked by an apostrophe.

Supplementary Table 4: Plasmids used in this study

**Transfection**		
**Identifier**	**Encoded protein**	**Origin/Citation**
pNT1112	ALFA-FLAG®-Vimentin	This study
pNT1067	Tom70-EGFP-ALFA	This study
pNT1066	pDisplay-HA-EGFP-ALFA-myc-TM	This study
pNT1004	NbALFA-mScarlet-I	This study
pEGFP-N1	EGFP	Clontech
pNT0942	EGFP-ALFA	This study
pNT0941	ALFA-EGFP	This study
**Bacterial expression**		
**Identifier**	**Encoded protein**	**Origin/Citation**
pNT1208	His_{14}-bdSUMO-FLAG®-HA-MBP-myc-ALFA	This study
pNT1111	His_{14}-bdSUMO-NbALFA	This study
pNT1177	ALFA-shGFP2-His_6	This study
pNT1050	His_{14}-bdSUMO-shGFP2-ALFA	This study
pNT1063	His_{14}-bdSUMO-ALFA-shsfGFP	This study
pNT1129	pSC YfgM-ALFA	This study
pSC-yfgM	*yfgM* (native promoter in pUA66)	*Götzke et. al.*[19]
pNT1196	His_{14}-bdSUMO-TST-bdNEDDD8-ALFA	This study
pNT1298	His_{14}-bdSUMO-NbCD62L-ALFA	This study

Supplementary Table 5: Antibodies

Antibody	Supplier	Order No
FluoTag®-X2 anti-ALFA AbberiorSTAR635P	NanoTag Biotechnologies	N1502-Ab635P-L
FluoTag®-X2 anti-ALFA IRDye800CW	NanoTag Biotechnologies	N1502-Li800-L
FluoTag®-X2 anti-Mouse IgG IRDye680RD	NanoTag Biotechnologies	N1202-Li680-L
anti-FLAG® M2	Sigma	F1804
anti-myc 9E10	SynapticSystems	#343 011
anti-HA F-7	SantaCruz	#sc-7392
Goat anti-rabbit IRDye680RD	Li-COR	#925-68071
Goat anti-mouse IRDye800CW	Li-COR	#925-32210
anti-Tubulin	SynapticSystems	#302 211
polyclonal serum recognizing MBP	SynapticSystems	pers. communication
goat anti-rabbit IgG, HRP-conjugate	Dianova	#GAR/IgG(H+L)/PO
anti-YfgM/PpiD	*Götzke et. al.*[19]	
anti-human CD3, clone UCHT1, FITC	BioLegend	#300405
anti-human CD19, clone HIB19, BV421	BioLegend	#302233
anti-human CD62L, clone DREG-56, APC	BD Biosciences	#561916

Supplementary Table 6: Primers

Name	Sequence	Reference
CaLl 01	GTC CTG GCT GCT CTT CTA CAA GG	*Olichon et. al.*[20]
CaLl 02	GGT ACG TGC TGT TGA ACT GTT CC	*Olichon et. al.*[20]
F1	TCT GGT GAT GCA TCT GAC AGC GAG GTG CAG CTG CAG GAG TCT GG	This study
R1	GTT TTC CCC AGT GGA TCC AGA AGT TTG TGG TTT TGG TGT CTT GGG	This study

Supplementary References

1. Hopp, T. P. *et al.* A short polypeptide marker sequence useful for recombinant protein identification and purification. *Bio/Technology* **6**, 1204–1210 (1988).
2. Wilson, I. A. *et al.* The structure of an antigenic determinant in a protein. *Cell* **37**, 767–778 (1984).
3. Evan, G. I., Lewis, G. K., Ramsay, G. & Bishop, J. M. Isolation of monoclonal antibodies specific for human c-myc proto-oncogene product. *Mol. Cell. Biol.* **5**, 3610–3616 (1985).
4. Schmidt, T. G. M. *et al.* Development of the Twin-Strep-tag® and its application for purification of recombinant proteins from cell culture supernatants. *Protein Expr. Purif.* **92**, 54–61 (2013).
5. Porath, J., Carlsson, J., Olsson, I. & Belfrage, G. Metal chelate affinity chromatography, a new approach to protein fractionation. *Nature* **258**, 598–599 (1975).
6. Hochuli, E., Döbeli, H. & Schacher, A. New metal chelate adsorbent selective for proteins and peptides containing neighbouring histidine residues. *J. Chromatogr.* **411**, 177–184 (1987).
7. De Genst, E. J. *et al.* Structure and properties of a complex of α-synuclein and a single-domain camelid antibody. *J. Mol. Biol.* **402**, 326–343 (2010).
8. Virant, D. *et al.* A peptide tag-specific nanobody enables high-quality labeling for dSTORM imaging. *Nat. Commun.* **9**, 1–14 (2018).
9. Braun, M. B. *et al.* Peptides in headlock - A novel high-affinity and versatile peptide-binding nanobody for proteomics and microscopy. *Sci. Rep.* **6**, 1–10 (2016).
10. Petukhov, M. *et al.* Design of stable alpha-helices using global sequence optimization. *J. Pept. Sci.* **15**, 359–365 (2009).
11. Hunter, M. R., Grimsey, N. L. & Glass, M. Sulfation of the FLAG epitope is affected by co-expression of G protein-coupled receptors in a mammalian cell model. *Sci. Rep.* **6**, 27316 (2016).
12. Schembri, L. *et al.* The HA tag is cleaved and loses immunoreactivity during apoptosis. *Nat. Methods* **4**, 107–108 (2007).
13. Hernan, R., Heuermann, K. & Brizzard, B. Multiple epitope tagging of expressed proteins for enhanced detection. *Biotechniques* **28**, 789–793 (2000).
14. Ross-Macdonald, P., Sheehan, A., Roeder, G. S. & Snyder, M. A multipurpose transposon system for analyzing protein production, localization, and function in Saccharomyces cerevisiae. *Proc. Natl. Acad. Sci. U. S. A.* **94**, 190–195 (1997).
15. Sharrock, R. A. & Clack, T. Heterodimerization of type II phytochromes in Arabidopsis. *Proc. Natl. Acad. Sci. U. S. A.* **101**, 11500–11505 (2004).
16. Graumann, J. *et al.* Applicability of tandem affinity purification MudPIT to pathway proteomics in yeast. *Mol. Cell. Proteomics* **3**, 226–237 (2004).
17. Lata, S., Reichel, A., Brock, R., Tampé, R. & Piehler, J. High-affinity adaptors for switchable recognition of histidine-tagged proteins. *J. Am. Chem. Soc.* **127**, 10205–10215 (2005).
18. Crowley, P. B. & Golovin, A. Cation-π interactions in protein-protein interfaces. *Proteins Struct. Funct. Genet.* **59**, 231–239 (2005).
19. Götzke, H. *et al.* YfgM is an ancillary subunit of the SecYEG translocon in Escherichia coli. *J. Biol. Chem.* **289**, 19089–19097 (2014).
20. Olichon, A., Schweizer, D., Muyldermans, S. & de Marco, A. Heating as a rapid purification method for recovering correctly-folded thermotolerant VH and VHH domains. *BMC Biotechnol.* **7**, 7 (2007).
