# Supplementary material for: The ALFA-tag is a highly versatile tool for nanobody-based bioscience applications
Source: Nat Commun. 2019 Sep 27;10:4403. doi: 10.1038/s41467-019-12301-7 (PMC6764986; doi:10.1038/s41467-019-12301-7)
Supplement: Supplementary file 3 — Reporting Summary [file 41467_2019_12301_MOESM3_ESM.pdf]

nature research

Corresponding author(s): Felipe Opazo
Steffen Frey

Last updated by author(s): Aug 9, 2019

Reporting Summary

Nature Research wishes to improve the reproducibility of the work that we publish. This form provides structure for consistency and transparency in reporting. For further information on Nature Research policies, see Authors & Referees and the Editorial Policy Checklist.

Statistics

For all statistical analyses, confirm that the following items are present in the figure legend, table legend, main text, or Methods section.

n/a	Confirmed	
☐	☒	The exact sample size (*n*) for each experimental group/condition, given as a discrete number and unit of measurement
☐	☒	A statement on whether measurements were taken from distinct samples or whether the same sample was measured repeatedly
☐	☒	The statistical test(s) used AND whether they are one- or two-sided<br>*Only common tests should be described solely by name; describe more complex techniques in the Methods section.*
☒	☐	A description of all covariates tested
☒	☐	A description of any assumptions or corrections, such as tests of normality and adjustment for multiple comparisons
☐	☒	A full description of the statistical parameters including central tendency (e.g. means) or other basic estimates (e.g. regression coefficient) AND variation (e.g. standard deviation) or associated estimates of uncertainty (e.g. confidence intervals)
☒	☐	For null hypothesis testing, the test statistic (e.g. *F*, *t*, *r*) with confidence intervals, effect sizes, degrees of freedom and *P* value noted<br>*Give P values as exact values whenever suitable.*
☒	☐	For Bayesian analysis, information on the choice of priors and Markov chain Monte Carlo settings
☒	☐	For hierarchical and complex designs, identification of the appropriate level for tests and full reporting of outcomes
☒	☐	Estimates of effect sizes (e.g. Cohen's *d*, Pearson's *r*), indicating how they were calculated

Our web collection on statistics for biologists contains articles on many of the points above.

Software and code

Policy information about availability of computer code

Data collection	Flow cytometry: Data collection was done using FACS Diva software (BD Biosciences).<br>ÄKTA chromatography: UNICORN™ 7.0.2 software (GE Healthcare Life Sciences)<br>STED and confocal images: Abberior Expert and Leica LAS AF v1.7.0<br>BESSY II electron storage ring operated by the Helmholtz-Zentrum Berlin, Germany
Data analysis	Flow cytometry: Data analysis was done using FlowJo (FlowJo LLC) and Microsoft Excel (Microsoft).<br>Data analysis: GraphPad Prism 5, 2012 GraphPad Software Inc.<br>Image processing: ImageJ, Fiji, Picasso Software (opensource https://www.biochem.mpg.de/5608779/Software), Adobe Photoshop.<br>Crystal structure: DIALS software, Phaser, Phoenix Autobuild and Coot. Structure refinement with Refmac5

For manuscripts utilizing custom algorithms or software that are central to the research but not yet described in published literature, software must be made available to editors/reviewers. We strongly encourage code deposition in a community repository (e.g. GitHub). See the Nature Research guidelines for submitting code & software for further information.

Data

Policy information about availability of data

All manuscripts must include a data availability statement. This statement should provide the following information, where applicable:

- Accession codes, unique identifiers, or web links for publicly available datasets
- A list of figures that have associated raw data
- A description of any restrictions on data availability

The atomic coordinates and structure factors have been deposited in the Protein Data Bank (http://www.wwpdb.org, code 6I2G). Primary data of graphs shown in Figures 4b, and Supplementary Figures 2, 6 and 7 are available in the Source Data file. All other datasets generated during and/or analyzed during the current study are available from the corresponding authors (F.O. and S.F.) on reasonable request.

Field-specific reporting

Please select the one below that is the best fit for your research. If you are not sure, read the appropriate sections before making your selection.

☒ Life sciences ☐ Behavioural & social sciences ☐ Ecological, evolutionary & environmental sciences

For a reference copy of the document with all sections, see nature.com/documents/nr-reporting-summary-flat.pdf

Life sciences study design

All studies must disclose on these points even when the disclosure is negative.

Sample size	No sample-size calculation was performed nor necessary
Data exclusions	No data was excluded of any analysis performed.
Replication	The few experiments that required a replication to prove the effect was not chance, were done as independent experiments and after analysis their standard deviation was displayed.
Randomization	This point is not relevant for our study. We designed and characterized molecular interactions in vitro
Blinding	This point is not relevant for our study. We designed and characterized molecular interactions in vitro

Reporting for specific materials, systems and methods

We require information from authors about some types of materials, experimental systems and methods used in many studies. Here, indicate whether each material, system or method listed is relevant to your study. If you are not sure if a list item applies to your research, read the appropriate section before selecting a response.

Materials & experimental systems

n/a	Involved in the study	
☐	☒	Antibodies
☐	☒	Eukaryotic cell lines
☒	☐	Palaeontology
☐	☒	Animals and other organisms
☐	☒	Human research participants
☒	☐	Clinical data

Methods

n/a	Involved in the study	
☒	☐	ChIP-seq
☐	☒	Flow cytometry
☒	☐	MRI-based neuroimaging

Antibodies

Antibodies used	monoclonal anti-FLAG®, clone M2 (Sigma: Cat# F1804)<br>monoclonal anti-myc, clone 9E10 (Synaptic Systems Cat#343011)<br>monoclonal anti-HA, clone F-7 (SantaCruz Cat# sc-7392)<br>polyclonal Goat anti-rabbit IRDye680RD (Li-COR Cat# 925-68071)<br>polyclonal Goat anti-mouse IRDye800CW (Li-COR Cat# 925-32210)<br>monoclonal anti-Tubulin (Synaptic Systems Cat# 302211)<br>polyclonal serum recognizing MBP (Synaptic Systems, personal communication)<br>polyclonal goat anti-rabbit IgG HRP-conjugate (Dianova Cat# GAR/IgG(H+L)/PO)<br>polyclonal anti-YfgM/PpiD (Developed by Götzke, H. et al. (2014) J. Biol. Chem. 289, 19089–19097.)<br>monoclonal anti-human CD3, clone UCHT1, FITC (BioLegend Cat# 300405)<br>monoclonal anti-human CD19, clone HIB19, BV421 (BioLegend Cat# 302233)<br>monoclonal anti-human CD62L, clone DREG-56, APC (BD Biosciences Cat# 559772)
Validation	polyclonal anti-YfgM/PpiD (Developed by Götzke, H. et al. (2014) J. Biol. Chem. 289, 19089–19097.)

Eukaryotic cell lines

Policy information about cell lines

Cell line source(s)	3T3 (DSMZ no. ACC 173), COS-7 (DSMZ no. ACC 60) and Hela (DSMZ no. ACC 57) cell lines were purchased from the German Collection for Microorganisms and Cell Cultures (DSMZ), Braunschweig, Germany.
Authentication	Authentication of cell line was done by the DSMZ cell bank.

Mycoplasma contamination	All cells were negative for mycoplasma in routine PCR assays.
Commonly misidentified lines (See ICLAC register)	*Name any commonly misidentified cell lines used in the study and provide a rationale for their use.*

Animals and other organisms

Policy information about studies involving animals; ARRIVE guidelines recommended for reporting animal research

Laboratory animals	2 Alpacas
Wild animals	n/a
Field-collected samples	n/a
Ethics oversight	All work involving animal experiments at NanoTag Biotechnologies complies with the relevant ethical regulations for animal testing and research. All experiments conducted do not require ethical approval, but are communicated to and accepted by the local authorities (LAVES Niedersachsen, Germany).

Note that full information on the approval of the study protocol must also be provided in the manuscript.

Human research participants

Policy information about studies involving human research participants

Population characteristics	Only one healthy human male was a participant of the study (age: 40 years, ethnicity: Caucasian).
Recruitment	One human research participants volunteered as blood donor. No specific selection criteria were applied other than health status and good physical condition on the day of the experiment.
Ethics oversight	Experiments involving human participants were approved by the ethical review committee of the University Medical Center Göttingen (case number 11/6/17) and were performed in accordance with relevant guidelines and regulations. An informed consent was obtained from the participants.

Note that full information on the approval of the study protocol must also be provided in the manuscript.

Flow Cytometry

Plots

Confirm that:

☒ The axis labels state the marker and fluorochrome used (e.g. CD4-FITC).

☒ The axis scales are clearly visible. Include numbers along axes only for bottom left plot of group (a 'group' is an analysis of identical markers).

☒ All plots are contour plots with outliers or pseudocolor plots.

☒ A numerical value for number of cells or percentage (with statistics) is provided.

Methodology

Sample preparation	Human peripheral blood mononuclear cells (PBMCs) were obtained from fresh blood using standard density gradient centrifugation. 60 mL of fresh blood were diluted with 40 mL of phosphate-buffered saline (PBS) supplemented with 1 mM EDTA and placed on top of a layer of CELLPURE Roti-Sep 1077 (Carl Roth, Karlruhe, Germany) in 50 mL LEUCOSEP tubes (Greiner Bio-One, Frickenhausen, Germany) and centrifuged at 800 x g for 20 minutes at room temperature. Subsequently, the PBMC-containing layer was collected and washed five times in cold PBS + EDTA to remove platelets.<br>CD62L-positive lymphocytes were isolated by passing approximately 2 x 10^7 PBMCs through an ALFA Selector resin loaded with an ALFA-tagged anti-human CD62L nanobody, followed by extensive washing with PBS supplemented with 1 mM EDTA 1 and 1 % (w/v) bovine serum albumin. Subsequently, bound cells were eluted in the same buffer containing 200 µM ALFA peptide.
Instrument	Data collection was done on a LSRII flow cytometer (BD Biosciences).
Software	Data collection was done using FACS Diva software (BD Biosciences).<br>Data analysis was done using FlowJo software (FlowJo, LLC).
Cell population abundance	Approximately 2 x 10^7 PBMCs were used for sorting. Of those, roughly 4 x 10^6 cells were eluted from the anti-CD62L-loaded ALFA selector resin. The eluted cells were stained with the indicated antibodies (anti-CD3-FITC + anti-CD19-BV421 + anti-CD62L-APC), extensively washed and analyzed by flow cytometry.

Gating strategy	A forward-scatter/side-scatter gate was used to gate on lymphocytes to exclude cell debris. Details on the gating strategy are given as Supplementary Figure 9.

☒ Tick this box to confirm that a figure exemplifying the gating strategy is provided in the Supplementary Information.
